# The Role of Skin Microbiota in Facial Dermatoses and Related Factors: A Narrative Review

**DOI:** 10.3390/ijms26188857

**Published:** 2025-09-11

**Authors:** Iva Ferček, Petar Ozretić, Lucija Zanze, Zoran Zoričić, Lorena Dolački, Rok Čivljak, Liborija Lugović-Mihić

**Affiliations:** 1 Department of Ophthalmology, Zabok General Hospital and Croatian Veterans’ Hospital, 49210 Zabok, Croatia; iva.fercek@gmail.com; 2 Laboratory for Hereditary Cancer, Division of Molecular Medicine, Ruđer Bošković Institute, 10000 Zagreb, Croatia; pozretic@irb.hr; 3 Family Physician Office, 10000 Zagreb, Croatia; lucijazanze15@gmail.com; 4 Department of Psychiatry, University Hospital Center Sestre Milosrdnice, 10000 Zagreb, Croatia; zoran.zoricic@kbcsm.hr; 5 School of Dental Medicine, University of Zagreb, 10000 Zagreb, Croatia; dolacki.lorena@gmail.com; 6 Department of Dermatovenereology, University Hospital Center Sestre Milosrdnice, 10000 Zagreb, Croatia; 7 Department for Respiratory Infections, University Hospital for Infectious Diseases “Dr. Fran Mihaljević”, 10000 Zagreb, Croatia; 8 School of Medicine, University of Zagreb, 10000 Zagreb, Croatia

**Keywords:** skin microbiota, microbiome, face, atopic dermatitis, acne vulgaris, contact dermatitis, seborrheic dermatitis, rosacea, psoriasis, immunology

## Abstract

Inflammatory facial dermatoses (atopic dermatitis [AD], acne vulgaris, contact dermatitis, seborrheic dermatitis, rosacea, perioral dermatitis, and demodicosis, etc.) often profoundly impact patients’ appearance and psychological well-being. In this narrative review, we wanted to present the current knowledge on the role of skin microbiota in common facial dermatoses. Skin keratinocytes are the primary producers of antimicrobial peptides (AMPs) and express Toll-like receptors (TLRs), which stimulate the T helper (Th1) immune response, with the production of interferon (IFN). They can also produce certain pro-inflammatory cytokines, namely IL-1β, IL-18, IL-6, IL-10, and the tumor necrosis factor (TNF). In healthy infants, the bacterial skin microbiota is predominantly composed of Firmicutes (genera *Staphylococcus* and *Streptococcus*), as well as Actinobacteria, Proteobactera, and Bacteroidota. The genera *Cutibacterium* and *Staphylococcus*, which have antimicrobial effects and compete with pathogens for nutrients/ecological niches, coexist symbiotically on the skin and can reduce the expression of TLR2 and TLR4. In patients with AD, lesional/non-lesional skin was found to have increased colonization by *Staphylococcus aureus* which reduces effector T lymphocytes’ ability to produce cytokines, such as IL-17A and IFN-γ, leading to decreased AMP production and impaired skin microbiota immune functionality. In patients with rosacea, the overexpression of TLR2 may stimulate elevated pro-inflammatory cytokine production (IL-8, IL-1β, and TNF-α, etc.), exacerbating the inflammatory response. Also, increased colonization by *Malassezia* yeasts triggers a Th2 immune response and cytokine secretion (IL-1α, IL-1β, IL-2, IL-4, IL-6, IL-8, IL-10, IL-12, TNF-α, beta-defensin, IFN-γ, nitric oxide, and histamine), and participates in signaling pathways. Insight into these factors may further improve clinical approaches to patients with facial dermatoses.

## 1. Introduction

Chronic inflammatory skin diseases represent common dermatological conditions, affecting large numbers of people. According to recent data in the literature, the prevalence of the most frequent skin diseases in Europe was 8.9% for fungal skin infections, 5.4% for acne, and 5.5% for atopic dermatitis (AD) or eczema [1]. Gaining knowledge on the exact pathogenesis of such diseases remains a diagnostic and therapeutic challenge for clinicians, as well as an economic burden on healthcare systems, due to the costs associated with their management [2]. Inflammatory diseases affecting the face are of particular significance, as they have a profound impact on the appearance of patients, their psychological well-being, and their overall quality of life. Non-infectious chronic facial dermatoses include AD, acne vulgaris, seborrheic dermatitis, rosacea, perioral dermatitis, periocular dermatitis, demodicosis, contact dermatitis, and, more rarely, psoriasis [3,4,5,6,7]. Connective tissue diseases and autoimmune disorders, such as discoid lupus erythematosus and dermatomyositis, may also manifest on the face. Furthermore, the face is the most common site for rejuvenation procedures (e.g., dermal filler injections and the use of laser technology), which can result in cutaneous changes, such as hypersensitivity reactions, infections, foreign body sensation, and subsequent erythematous or hypo-/hyperpigmented lesions [3,8]. The simultaneous occurrence of these conditions is possible, further complicating accurate diagnosis and effective treatment.

The composition of the skin microbiome and its deviations are important factors in the pathogenesis of inflammatory facial dermatoses [9,10,11]. The human microbiota refers to all microorganisms residing on or within our bodies in specific microenvironments, such as the skin, gut, or oral cavity [12,13,14,15,16,17]. These microorganisms contribute to the maintenance of bodily homeostasis and the modulation of the host immune response. Alterations in the composition of the microbiota have a potential role in disease development [9,10]. The terms “microbiome” and “microbiota” are often used interchangeably; however, the microbiome is a broader concept that encompasses the totality of microorganisms (microbiota), their genomes, and environmental factors [11].

A healthy skin microbiota is composed mainly of four bacterial phyla: Actinobacteria (36–52%), Firmicutes (24–34%), Proteobacteria (11–16%), and Bacteroidetes (6–9%). Key genera include *Cutibacterium, Staphylococcus,* and *Corynebacterium*, which dominate different skin environments; *Cutibacterium* is prevalent in oily regions, *Staphylococcus* and *Corynebacterium* in moist areas, and higher diversity is found in dry areas [15,16]. In addition to bacteria, the microbiome also includes fungi (primarily *Malassezia*), archaea (like Thaumarchaeota, ~4%), viruses, and mites [17]. This balanced microbiome supports the skin barrier function, regulates the pH, and educates the immune system, providing a defense against pathogens and maintaining the hydration and resilience of the skin. Disruptions to this balance can lead to impaired barrier function and skin disorders [18].

As part of the normal skin microbiota, several species of *Staphylococcus* are commonly present. *Staphylococcus epidermidis* (*S. epidermidis*), for example, is a very frequent skin inhabitant and, in some areas of the skin, it makes up more than 90% of the aerobic flora. However, other species, such as *Staphylococcus aureus* (*S. aureus*), a gram-positive pathogenic bacterium, are not usually present in healthy skin microbiota; *Staphylococcus aureus* is only found in certain areas, for e.g., the nose and perineum, and may express various toxin factors and proteases, see [13]. *S. aureus* possesses various cell wall proteins and secreted factors, which may adhere to and impair the skin barrier through physical, chemical, and inflammatory mechanisms. For instance, *S. aureus* colonization in patients with AD involves changes to the skin’s epidermal barrier, increased bacterial adhesion, impaired bacterial clearance, and a decrease in the innate immune response. According to research data, *S. aureus* colonization is associated with AD pathogenesis, disease flares, and disease phenotypes [13].

**Figure 1:**
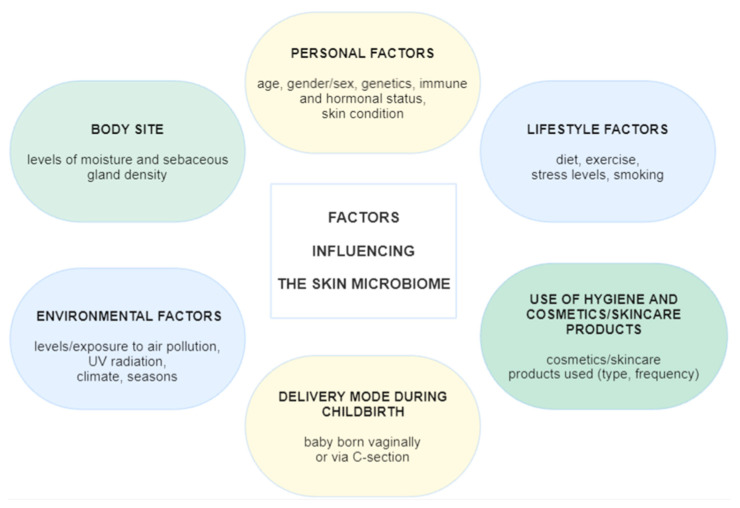
Factors influencing the skin microbiome.

However, there are various factors that influence the role of the skin microbiota in facial dermatoses (Figure 1). The diversity of the human microbiome is influenced by a complex interplay of external and internal factors. External factors include environmental conditions (e.g., climate, seasonal factors, and pollution), lifestyle choices (such as diet, hygiene practices, and physical activity), the use of pharmaceuticals and personal care products (notably antibiotics and antiseptics), occupational exposures, and interactions with other individuals or animals. Internal factors encompass a person’s age, sex, hormonal influences, genetic background, immune system function, and overall health status [4,10]. These variables collectively contribute to the composition, stability, and temporal dynamics of the microbiome across different stages of life.

The human skin microbiome is diverse and is influenced by factors like the body site, and, as such, it is a complex and dynamic community of microorganisms, which are not randomly distributed, but that depend on the skin’s biophysical properties, like the pH, moisture levels, lipid content, and exposure to environmental factors [4,6,14]. For instance, sebaceous areas are rich in lipids and promote lipophilic organism growth, such as *Cutibacterium acnes* (*C. acnes*), which metabolizes sebum triglycerides into free fatty acids. Also, moist areas, like the axillae, mostly harbor *Corynebacterium* species, and dry regions predominantly host *S. epidermidis* and other resilient bacteria. In sebaceous areas, fungi (e.g., the genus *Malassezia*) are also common, while other microbial communities vary across different body sites and skin types [14].

The skin microbiome composition may also be related to a person’s topical treatment use, hormonal status (especially in females), or seasonal variations, among other factors [4,6,14]. Hormonal fluctuations, for example, may particularly impact the skin microbiome during puberty and the menopause (e.g., shifts in skin microbial composition potentially lead to conditions like acne or eczema). The increase in hormones during puberty (like androgens) increases sebum production, which favors certain microorganism growth like *C. acnes*, leading to skin dysbiosis and potentially contributing to acne. Also, hormonal changes during the menopause (e.g., decreased estrogen) may influence the skin microbiome, such that skin hydration and elasticity are affected, potentially increasing the susceptibility to certain dermatoses. In addition, there are other types of hormonal influences on the skin, for e.g., during psychological stress, higher cortisol may trigger inflammation and impact the skin’s barrier function, potentially leading to dermatoses. Hormonal changes may also influence gut microbiomes, which affect skin conditions and dermatoses. (The skin microbiome is interconnected with the gut microbiome through the gut–skin axis.) Certain skin microbes may also produce or interact with hormones; some gut bacteria may produce short-chain fatty acids and neurotransmitters that influence a person’s health and skin condition. 

In addition, seasonal shifts can alter the balance of microorganisms on skin [13,14]. In particular, cold weather often leads to skin dryness and potentially impairs the skin barrier, while warmer months can increase sweat and oil production, which can impair the skin microbiome and potentially lead to skin lesions. Also, the skin microbiome composition undergoes significant changes with aging, influenced by shifts in host factors like skin lipids, natural moisturizing factors (NMFs), sebaceous gland activity, and antimicrobial peptides (AMPs) [6]. According to research data, bacterial diversity increases with age in some localizations (the forearms, face, and buttocks, etc.), while the abundance of some bacteria on all skin sites decreases with age (genera *Cutibacterium* and *Lactobacillus*) (correlating with a decrease in the sebaceous gland size and lipid production). On the other hand, genera like *Streptococcus* and *Anaerococcus* have shown site-specific increases, likely driven by changes in skin lipids and other host factors. Age-associated increases in NMFs and AMPs positively correlate with some bacterial genera, like *Corynebacterium* and *Finegoldia*. Also, race and ethnicity significantly impact microbiome composition, with differences that also depend on age. Also, there is an association between variability in microbial taxa and inequitable environmental and social factors, for e.g., specific taxa like *Bifidobacterium* and *Lactobacillus* are more present in racial groups with higher breastfeeding rates. Other differences correlate with factors like diet and hygiene.

The body’s different skin localizations/regions typically involve/host a variety of microorganisms that adapt to their specific microenvironments, including temperature, sebum production, and sweat levels [4,10,13,14]. Commensals have a regulatory role in maintaining host health by preventing invasion from pathogenic microorganisms, influencing host immune responses; *S. epidermidis*, for instance, produces antimicrobial substances, while *C. acnes* utilizes skin lipids to generate short-chain fatty acids, supporting anti-inflammatory effects and host homeostasis. Also, the host impacts the microbiome composition through the immune system; for e.g., the host contributes to establishing/shaping the microbial community through the production of epidermal keratinocyte AMPs from the sebaceous glands, lipid antimicrobials, and cytokines, etc. [13]. In addition, fungal and viral components of the microbiome also have important roles in skin health and disease. The *Malassezia* species of fungi is frequently present on healthy skin, but their uncontrolled growth can lead to dermatoses like seborrheic dermatitis and pityriasis versicolor. Viral commensals generally remain latent in the skin, but can become pathogenic during immunosuppression or barrier disruption (e.g., human papillomaviruses and herpesviruses). So, the network and interplay between microorganisms, the host immune system, and external factors influence the balance between skin health and disease [14].

Finally, skin dysbiosis is implicated in a range of dermatoses, including facial dermatoses. In dermatoses like AD, dysbiosis involves an overgrowth of *S. aureus*, which triggers skin inflammation and impairs the epidermal barrier. The dysfunctional skin microbial barrier and T cell-mediated immune response are crucial to the development of dermatoses. Acne vulgaris is characterized by changes in the abundance and activity of *C. acnes*, wherein some strains can overproduce pro-inflammatory lipids and contribute to follicular inflammation [14]. Also, psoriasis and rosacea involve various skin cell changes, including T cell infiltration and vascular proliferation, which may be related to the skin microbiome.

For this narrative review, we analyzed data from the literature published in prominent medical databases, such as the Web of Science (WOS), Scopus, and PubMed, during the period between 2007 and 2025. The search was conducted using the following terms: “skin microbiome”; “microbiome”; “face”; “atopic dermatitis”; “acne vulgaris”; “contact dermatitis”; “seborrheic dermatitis”; “rosacea”; “psoriasis”; and “immunology”. Here, we present key knowledge and results from the recent studies on the topic of the role of the skin microbiome in facial dermatoses and related factors. Thus, this article is based exclusively on previously published literature.

## 2. The Role of the Skin Barrier

Understanding the structure of the skin is essential for comprehending the skin microbiome and its interactions with the host [4,10,18,19]. The skin consists of three layers, the epidermis, dermis, and deeper subcutaneous tissue (hypodermis), which together function as a barrier. This barrier is composed of physical, chemical, microbiological, and immunological factors that defend the body against external pathogens. Structurally, the epidermis comprises five distinct cellular layers: the stratum basale, stratum spinosum, stratum granulosum, stratum lucidum, and stratum corneum. Basal keratinocytes are firmly attached to the underlying basement membrane, while some daughter cells migrate through all layers of the epidermis via asymmetric mitosis, supporting the self-renewal of the epidermis during a process known as terminal differentiation [18]. The tightly interconnected corneocytes of the stratum corneum, which are continuously shed from the skin’s surface, are a key component of the skin barrier. The entire stratification cycle is strictly regulated to prevent skin damage [18]. The shedding of corneocytes is controlled by kallikrein-related proteases that are secreted by keratinocytes in the stratum corneum, along with counter-regulatory protease inhibitors. The balance between protease activity and protease inhibitors is crucial for the desquamation process across different body sites, which provides increased resilience to areas subjected to greater mechanical stress, such as the palms and soles [19].

Alongside corneocytes, the stratum corneum consists of an extracellular lipid-rich matrix that includes free fatty acids, cholesterol, and ceramides. Because of this composition, the entire structure of the stratum corneum is often described as a “brick and mortar” model, wherein corneocytes act as the bricks and lipids serve as the mortar, contributing to the formation and maintenance of the skin’s permeability barrier [20]. Filaggrin, a histidine-rich protein produced in keratohyalin granules within the granular cell layer, plays a crucial role in the formation of the skin barrier. Profilaggrin, the precursor of filaggrin, is encoded by the FLG gene located on chromosome 1q21 and represents a major genetic risk factor for the development of AD, where loss-of-function mutations occur. During keratinocyte differentiation into corneocytes, filaggrin aggregates keratin into macrofibrils within corneocytes and is subsequently degraded into water-soluble molecules that constitute the NMF. These NMFs are involved in regulating the skin pH and transepidermal water loss [19]. All of these factors may be related to skin microbiome characteristics in the context of the development of facial dermatoses.

The condition of the epidermal barrier and any disturbances to it may be related to factors like the abundance of skin microorganisms. It should be mentioned that commensal microorganisms on the skin interact with the host, performing key functions like strengthening the skin barrier, immune modulation, and overcoming potential pathogens. For example, *S. epidermidis* produces AMPs (like *phenol-soluble modulins*), which inhibit the pathogenic colonization of bacteria like *S. aureus*. Similarly, *C. acnes* may regulate the skin’s pH by hydrolyzing sebum triglycerides, thus contributing to an acidic environment, which is unfavorable to pathogens. Commensals also influence physical barrier integrity by promoting keratinocyte differentiation and lipid production, which are important for stratum corneum formation [12,14]. Skin dysbiosis, with concomitant changed skin microbiome function, may affect the skin microbial barrier by inducing water loss and abnormal lipid metabolism, leading to skin inflammation and dermatoses [12,13]. It is also important to note that some microorganisms release toxins that can impair and dissolve the stratum corneum, reducing the barrier function and allowing their entry into the bloodstream. An example is *S. aureus*, which secretes the alpha-toxin and many proteases (including serine proteases and kallikrein), and imbalances in these protease activities may dissolve the skin’s stratum corneum, leading to impairments in the skin barrier function. *S. aureus* also produces lipoteichoic acid, which causes impairments to the skin barrier by inhibiting the expression of epidermal barrier proteins, like fibroin and loricrin [13].

## 3. The Role of the Immune System

The immune system also functions as a skin barrier and consists of both nonspecific innate immunity and highly specific adaptive immunity. Various immune cells in the skin secrete cytokines and chemokines that modulate the host’s skin immune response [21,22,23,24,25]. The innate immune system, as the first line of defense, responds rapidly and nonspecifically to all foreign pathogens by activating recognition systems and effector mechanisms. In contrast, the adaptive immune response is slower but specifically targeted against particular pathogens and possesses the ability to develop and maintain an immunological memory. When the innate immune response is insufficient to eliminate a pathogen, the adaptive immune response is activated as a stronger and more specific reaction to antigens.

Keratinocytes are the primary producers of AMPs in the skin and express Toll-like receptors (TLRs) on their surface and within endosomes, which stimulate the immune response of T helper cells (Th1) and the production of interferon (IFN). They can also produce pro-inflammatory cytokines, such as interleukins IL-1β, IL-18, IL-6, IL-10, and the tumor necrosis factor (TNF). Skin AMPs are an essential component of the innate immune system. The main families of AMPs include cathelicidins, human β-defensins, S100 proteins, and ribonucleases, which can be found in keratinocytes, eccrine gland cells, mast cells, phagocytes, and sebocytes [22]. Some AMPs are constitutively present in the skin, while the production of others is induced upon contact with pathogens. In addition to microorganisms, pro-inflammatory cytokines and the TNF can also stimulate AMP production. Numerous studies have investigated the potential role of AMPs in the development of inflammatory skin diseases, particularly psoriasis, AD, acne, and rosacea [23,24,25,26].

Langerhans cells, which serve as antigen-presenting cells, are the principal immune cells of the skin located within the epidermis, while various subpopulations of dendritic cells, macrophages, and several types of T cells reside in the dermis. Langerhans cells represent functionally “immature” dendritic cells; they express lower levels of Major Histocompatibility Complex class II (MHC II) molecules on their surface and stimulate T lymphocytes less effectively compared to “mature” dendritic cells, such as those found in peripheral blood. According to recent studies, Langerhans cells are not involved in promoting inflammatory processes, but rather contribute to tolerogenic responses through IL-10 production and the induction of CD4+ regulatory T cells and tolerogenic CD8+ T cells [27]. During health and homeostasis, the microbiome exists in symbiosis with its host and participates in maintaining the skin barrier. However, when the barrier is disrupted by unfavorable intrinsic or extrinsic factors, the microbiota can activate both innate and adaptive immune responses to restore homeostasis. The skin microbiota modulates the expression of various innate factors produced by keratinocytes and sebocytes, including IL-1α, complement components, and AMPs [28]. Thus, various factors may influence the role of skin microbiota during the development of facial dermatoses.

In addition, microorganisms that enter the bloodstream may further activate molecular signals, leading to immune network and skin inflammatory responses; for e.g., *Malassezia* stimulates skin dendritic cells to release cytokines IL-12 and IL-23, which differentiate into Th17 cells and produce pro-inflammatory mediators (IL-17, IL-22, TNF-α, and IFN-α). The inflammatory response may also be influenced by abnormal protein folding, cellular stress, interferon activation, and changed/disordered nuclear factor-κB, among other factors and mechanisms [12,13]. Thus, the interaction between the skin microbiome and the host immune system is important in regard to the condition of the skin. As mentioned before, commensals, through host pattern recognition receptors like TLRs and nucleotide-binding oligomerization domain-like receptors, modulate immune responses. These interactions provide a balance between immune tolerance to commensals and activation against invading pathogens. Thus, *S. epidermidis* may stimulate the keratinocyte TLR2 receptor upon the induction of AMP production, while also promoting regulatory T cell (Treg) activity that prevents excessive inflammation. Thus, the microbiome has immune-regulating functions, which are key for maintaining a balanced skin immune status and reducing potential inflammatory/autoimmune conditions [14].

## 4. The Microbiome of Healthy Skin

The skin has the second highest bacterial density after the gut and provides a habitat for eighteen distinct bacterial phyla, with four phyla predominating: Actinobacteria, Firmicutes, Proteobacteria, and Bacteroidota. More than 200 bacterial genera have been identified, with *Corynebacterium*, *Cutibacterium*, and *Staphylococcus* being the most prevalent [29]. Certain bacterial species preferentially colonize specific skin sites, which differ in response to certain characteristics, such as ultraviolet radiation exposure, temperature, moisture level, sebum content, oxygen availability, and pH. Nevertheless, the skin microbiome is dynamic and is influenced by external environmental factors and lifestyle habits (e.g., occupation, climate, use of cosmetics and antibiotics), as well as intrinsic factors, such as the person’s age and sex.

Considering the specific characteristics of the skin in relation to bacteria, the skin surface is typically mildly acidic (a pH of around 5.6) and dry. Seborrheic areas of the skin (such as the face, chest, and back) are dominated by lipophilic bacteria of the genera *Cutibacterium* and *Staphylococcus*, which can metabolically utilize sebum and tolerate low pH values [29,30]. These skin regions are also characterized by the lowest microbial diversity [31]. Bacteria of the genus *Cutibacterium* belong to the phylum Actinobacteria and require anaerobic growth conditions; therefore, they can also be isolated deeper in the dermis, specifically within pilosebaceous units, which are areas rich in sebum. These bacteria produce lipases that facilitate the conversion of triglycerides into short-chain fatty acids, which may potentially contribute to the development of skin inflammation [18]. Within the seborrheic skin microbiota, the genus *Staphylococcus* is the second most abundant and belongs to the phylum Firmicutes. Furthermore, bacteria of the genus *Staphylococcus* (such as *S. aureus* and *S. epidermidis*) are able to tolerate the low pH of sebaceous skin and produce lipases to exploit lipid-rich substrates. The genera *Cutibacterium* and *Staphylococcus* coexist symbiotically on the skin, and their antimicrobial defense is based on the direct inhibition of pathogenic growth. They compete with pathogens for nutrients and ecological niches and can desensitize and reduce the expression of receptors TLR2 and TLR4, which are important components of the innate immune system and the first line of defense against pathogenic microorganisms [32].

The specificity of bacterial colonization is observed according to distinct skin regions. Moist areas of the skin (such as the folds of the elbows, knees, and groin) are predominantly colonized by bacteria that tolerate humid conditions well, including the genera *Staphylococcus* and *Corynebacterium* [29,30]. Bacteria of the genus *Corynebacterium* (phylum Actinobacteria) are present across all skin sites, particularly in moist areas. Most species within the skin microbiota do not cause specific diseases in homeostatic conditions; however, in certain circumstances, they may participate in activating the skin immune response and IL-23-dependent inflammatory pathways [33]. Bacteria from the phylum Proteobacteria dominate dry skin areas (such as the volar forearm and palm) and represent the largest bacterial phylum, exhibiting considerable metabolic diversity. This group includes commensals, pathogenic genera, and numerous free-living bacteria. Human pathogenic representatives of this phylum include the genera *Brucella*, *Rickettsia*, *Bordetella*, *Neisseria*, *Escherichia*, *Shigella*, *Salmonella*, *Yersinia*, and *Helicobacter* [29,30,34].

In regard to the pathology of facial and eyelid skin, the mites of the genus *Demodex* are particularly significant. This ectoparasite is part of the normal microbiome in healthy individuals, but is more prevalent in patients with chronic blepharitis and rosacea, suggesting its potential role in disease development [35]. Demodicosis refers to infestation by *Demodex* mites on the skin. Two species inhabit human skin: *Demodex folliculorum* (*D. folliculorum*) and *Demodex brevis* (*D. brevis*). *Demodex* is a saprophytic mite belonging to the family Demodicidae (class Arachnida, order Acarina), measuring up to 0.4 mm in length, making them visible only under a microscope [36]. *Demodex* resides at the base of hair follicles within the pilosebaceous units of the facial skin, including the cheeks, nose, chin, forehead, temples, eyelashes, and eyebrows, as well as the scalp, neck, and ears. *D. folliculorum* is more commonly found on the face, whereas *D. brevis* is more frequently located on the neck and chest [37]. Research indicates that *Demodex* induces immunotolerance, allowing them to persist on healthy skin, but they can also trigger inflammation via TLR2, activating the immune system, increasing the production of the antimicrobial peptide LL-37, and promoting angiogenesis [38]. The detection of *Demodex* mites on the skin is performed using various techniques, such as skin scrapings, the cellophane tape method, plucking entire eyelashes, and a biopsy, followed by a microscopic examination. Currently, molecular methods are also available for detecting *Demodex*, which have demonstrated sufficient sensitivity; however, isolation techniques remain relatively complex and costly [39].

In addition to the aforementioned bacteria and parasites, fungi and viruses are also present on the skin. The study of viruses has been enabled by the application of shotgun metagenomic sequencing. The viral component of the microbiome, known as the virome, consists of bacteriophages that infect bacteria, as well as eukaryotic viruses and endogenous retroviruses that infect human cells. However, research on the human skin virome is limited due to the complexity of the isolation procedures and biostatistical analysis, although existing data suggest that the human virome depends on the skin microenvironment [30,40]. The fungal microbiota of the skin (mycobiota) is predominantly composed of yeasts from the class Malasseziomycetes and the genus *Malassezia*, including species such as *Malassezia restricta*, *Malassezia globosa*, and *Malassezia sympodialis*, which are found across all skin sites [41]. In children, whose sebaceous gland activity is lower than in adults, fungal communities are more diverse and include genera such as *Aspergillus*, *Epicoccum*, and *Phoma* [42]. Similarly, on the skin of the feet, genera such as *Malassezia*, *Cryptococcus*, *Aspergillus*, *Rhodotorula*, *Epicoccum*, *Saccharomyces*, *Candida*, *Epidermophyton*, *Microsporum*, and *Trichophyton* are present [43]. In healthy skin, *Malassezia* exists as a commensal organism and interacts benignly with keratinocytes and the immune system. The host recognizes fungi either directly through interactions between fungal cell wall components and membrane-bound pattern recognition receptors (PRRs), or indirectly via soluble metabolites that are released by yeasts, such as *Malassezia* [44]. However, the precise roles of these receptors (and other PRRs) in initiating inflammation and adaptive immunity remain unclear. *Malassezia* yeasts secrete lipases that degrade sebum into long-chain fatty acids, which explains their higher prevalence in sebum-rich areas supplied by sebaceous glands, although they are also found on all of the other skin sites. During this process, *Malassezia* produces unsaturated fatty acids, mainly oleic acid, that may play a role in compromising the skin barrier. This highlights the key role of lipases in the pathogenesis of dermatoses associated with *Malassezia* yeasts, such as seborrheic dermatitis (including dandruff), AD, and pityriasis versicolor, because lipases enable fungal survival on the skin and modulate the immune response [45].

The skin microbiome undergoes two major phases of change from birth onward: it is first influenced by the newborn’s mode of delivery, and later by hormonal changes during puberty. The skin of newborns is immediately exposed to environmental microorganisms that begin colonizing it (the early colonization process), which is significant for the development of tolerance to skin commensals and potentially influences the later onset of inflammatory skin diseases [12,46].

In preterm neonates, the bacterial skin microbiota is dominated by bacteria from the phyla Firmicutes (genus *Staphylococcus*) and Bacteroidota (genus *Flavobacterium*), with a higher representation of Firmicutes and a lower representation of Proteobacteria compared to the skin microbiota of full-term neonates [47,48,49,50,51,52,53]. In healthy infants, the skin microbiota is predominantly composed of Firmicutes (genera *Staphylococcus* and *Streptococcus*), followed by Actinobacteria, Proteobacteria, and Bacteroidota [46,47,48]. The skin microbiome continues to evolve throughout childhood and adolescence, in accordance with hormonal changes that promote a shift in the microbial composition towards a predominance of lipophilic microorganisms, particularly *C. acnes* and the yeast, *Malassezia restricta* (*M. restricta*) [49]. In adults, the skin microbiome remains relatively stable, with compositional differences depending on the specific skin site. Skin aging is influenced by intrinsic and extrinsic factors, the most significant of which is ultraviolet radiation exposure. Studies have shown that in women over 55 years of age (associated with decreased sebaceous gland activity), there is a reduction in the abundance of Firmicutes and a concurrent increase in Proteobacteria compared to women under 30 years of age [50]. Increased bacterial biodiversity has also been observed in mature skin, particularly in sun-exposed areas, while aging is associated with a decrease in the abundance of *M. restricta* and an increase in the representation of other fungal genera on the cheeks [18].

## 5. Methods for Studying the Microbiome

The study of the skin microbiome progressed significantly after the sequencing of the human genome, followed by investigations into its variations and associations in regard to specific diseases, as well as the ways in which the microbiome contributes to human health and disease [4,29,30,31]. Early research on the human microbiome focused on identifying microorganisms colonizing various parts of the body to gain insight into the complex interactions between the human host and the microbiome, and to explore potential contributions to the development of new treatments and health-promoting interventions. Two culture-independent methods have been used for microorganism identification in microbiome research: Terminal Restriction Fragment Length Polymorphism (T-RFLP) and gene sequencing. Both methods rely on the principle that the sequence of deoxyribonucleic acid (DNA) varies within conserved regions of the 16S ribosomal ribonucleic acid (rRNA) gene, depending on the bacterial species. In regard to both techniques, the initial step involves isolating bacterial DNA from the sample, followed by the Polymerase Chain Reaction (PCR), and subsequent fragment analysis and sequencing [4,31].

Groups of similar sequences, i.e., variable regions of the 16S rRNA gene that are highly similar but not identical, are referred to as Operational Taxonomic Units (OTUs). Additionally, alpha diversity (the richness and diversity of bacterial taxonomic groups within an individual) and beta diversity (the diversity of bacterial taxonomic groups between individuals) are analyzed to assess sample clustering, based on microbiota composition similarity [4,31].

In addition, an ASV (Amplicon Sequence Variant) is a high-resolution method for microbiome analysis that is used in research for the identification and analysis of microbial communities. This method distinguishes itself from traditional OTU-based methods by resolving sequence variations at the single-nucleotide level. Thus, this diagnostic method provides a more precise and reliable assessment of skin microbial composition and its diversity.

## 6. The Role of the Skin Microbiome in the Most Common Inflammatory Skin Diseases

The skin microbiome has been primarily studied in patients with AD, seborrheic dermatitis, psoriasis, and acne, and, to a lesser extent, in patients with rosacea, contact dermatitis, and other inflammatory skin diseases [51,52,53,54,55,56].

### 6.1. The Skin Microbiome in Patients with Atopic Dermatitis

**Figure 2:**
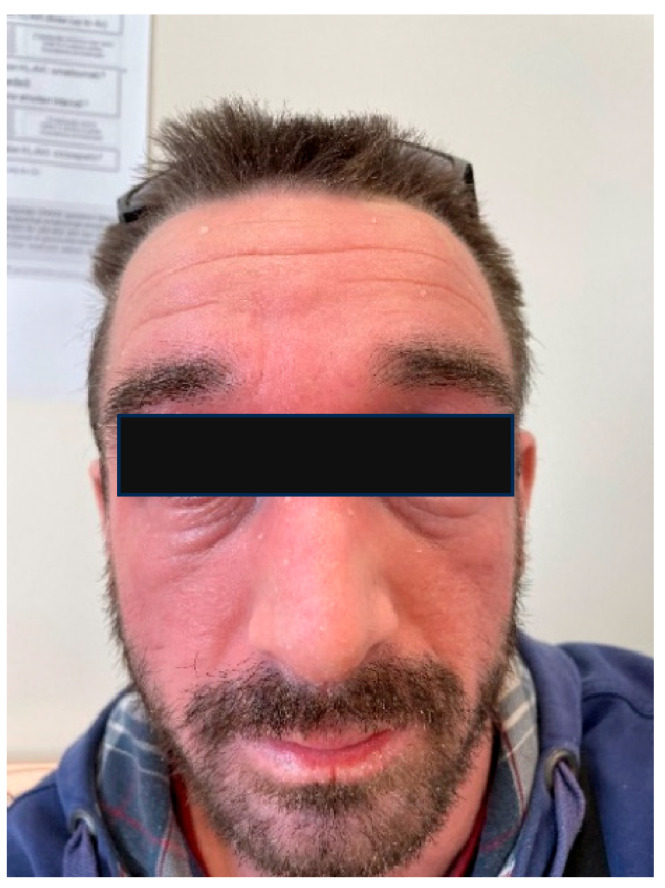
Atopic dermatitis—facial lesions.

AD is a chronic, relapsing dermatosis, characterized by eczema and often associated with genetic factors and allergy (Figure 2). The etiopathogenesis of AD is complex and is not yet fully understood; it involves the interaction of genetic, immune, and environmental factors that lead to damage to the stratum corneum and dysregulation of the immune response, in which the microbiota plays an important role [57,58,59,60]. A critical component of these complex interactions is the protective skin barrier, known as the epidermal barrier. Genetic mutations in the filaggrin gene (one of the most important epidermal proteins) are observed in approximately half of the patients with moderate-to-severe AD and are associated with early disease onset [57]. It is believed that epidermal barrier disruption triggers an acute inflammatory response following the activation of the Th2 pathway, characterized by elevated Th2 cytokine levels (such as IL-4, IL-5, and IL-13) and an increase in the total serum IgE and the number of circulating eosinophils, whereas chronic phases are characterized by Th1/Th17 responses. In patients with filaggrin gene mutations, lower levels of NMFs are often found in the stratum corneum. NMFs are crucial for maintaining the hydration of the deepest layers of the stratum corneum and preserving the skin pH balance. They may also serve as a nutrient source for certain bacteria that utilize histidine as a carbon source, such as gram-positive anaerobic cocci (GPAK) [58].

Among dermatoses, most available data is on the skin microbiome in AD, as it has been the subject of numerous studies that have revealed that AD is characterized by an imbalanced skin microbiome, i.e., dysbiosis [59,60,61,62,63,64]. A reduction in bacterial diversity and an increased prevalence of *S. aureus* instead of *S. epidermidis* have been found in the skin lesions of AD patients; the ratio of these bacteria also correlates with the AD severity. In addition, the proportion of *S. aureus* is much higher in areas of skin lesions than in adjacent, unaffected skin, which suggests that *S. aureus* plays a role in AD inflammatory processes. Coagulase-negative staphylococci, such as *S. epidermidis*, play a role in immune modulation; thus, a deficiency in these bacteria reduce the skin’s innate defense against *S. aureus*, which has been observed in patients with chronic AD. Structural differences in bacterial colonization by coagulase-negative staphylococcal strains and their reduction may lead to reduced AMP production and weaker immune functioning of the skin microbiome.

Most previous studies of the bacterial microbiota in AD patients have identified increased colonization by *S. aureus*. A higher abundance of this bacterium has been found not only on lesional skin of AD patients compared to healthy controls, but also compared to the non-lesional skin of affected individuals [30,52,59,61,62,63,64,65,66]. However, some studies suggest that *S. aureus* may serve as a disease marker rather than a key pathogenic factor [65,66]. For example, in individuals with Netherton syndrome (characterized by severe erythema and pathophysiological features similar to AD), there is no increase in *S. aureus* colonization on non-lesional skin, suggesting that atopic manifestations may not be solely due to an increase in the abundance of this bacterium. This is further supported by the observation that antibiotic therapy in AD patients often does not lead to clinical improvements [64].

In addition to the prevalence of *S. aureus*, other *Staphylococcus* species, particularly *S. epidermidis*, are more abundant in AD [52,59,67]. Increased *S. aureus* colonization correlates with more severe disease, whereas *S. epidermidis* is more prevalent in milder forms of AD [68]. Studies measuring alpha diversity have shown a reduction in bacterial biodiversity on the skin of AD patients [60,61,62,63], with a decrease in the relative abundance of bacterial genera, such as *Streptococcus* [59], *Cutibacterium*, and *Corynebacterium* [52,59]. Patients with chronic AD also exhibit an impaired innate defense against *S. aureus*. Moreover, *S. aureus* reduces the ability of effector T lymphocytes to produce cytokines, such as IL-17A and IFN-γ, leading to a decrease in AMP production and impaired immune functionality of the skin microbiota [69,70]. Furthermore, metabolites produced by skin commensals (e.g., GPAK) can lower the skin pH and enhance antimicrobial activity, resulting in reduced adhesion and the growth of *S. aureus* in human keratinocytes [58].

According to data in the literature, *S. aureus* may produce many surface molecules like fibronectin binding proteins, clumping factors A and B, and iron-regulated surface determinant protein A for adhering to the stratum corneum [13]. In AD skin lesions, Th2 cytokines (e.g., IL-4 and IL-13), which predominate in AD, and increased fibronectin/fibrinogen expression could increase the adhesion of *S. aureus* to the stratum corneum. The overexpression of IL-4 and IL-13 on AD skin causes the downregulation of cathinone (LL-37) and human β-defensin 3. Also, in AD patients, decreased AMP levels could increase the risk of long-term *S. aureus* skin colonization. On average, the skin of AD patients often has a weak alkaline pH, which in the stratum corneum could decrease sphingolipids levels (an important cell membrane component). Low sphingolipid expression can cause instability in stratum corneum cell membranes, thereby leading to increased transepidermal water loss and changes to the pH value and serum IgE level, and may lead to the activation of cytokines [thymus and the activation of cytokines (TARC)/CCL17) and eosinophils], which leads to the acceleration of *S. aureus* colonization. Natural moisturizing factors, like filaggrin and filaggrin degradation products (FDPs), are crucial for maintaining the skin pH and inhibiting skin *S. aureus* growth. In addition, lower FDP levels are linked to AD severity and induce strong adhesion of *S. aureus* to KC [13]. It should also be mentioned that *S. aureus* releases many virulence factors, mainly Staphylococcal enterotoxins, which are characterized by super antigenic properties, like SEA, SEB, and the toxic shock syndrome toxin. Thus, *S. aureus* superantigens may activate many T cell clones and produce factors with strong immune responses; they may trigger IgE-mediated mast cell degranulation, thus increasing the amount of Th2 cytokines; superantigens may bind to Langerhans cells/macrophages human leukocyte antigen (HLA)-DR and then stimulate the production of cytokines IL-1, TNF-α, and IL-12, resulting in AD exacerbation/recurrence. Also, *S. aureus* may induce keratinocyte apoptosis and release thymic stromal lymphopoietin, which could mediate the itch sensation and induce the activation of skin dendritic cells and Th2 recruitment. However, while *S. aureus* is commonly recorded at a higher abundance in AD lesions, some studies suggest that certain *S. aureus* strains might even have a protective effect in early life by modulating the immune system. As illustrated above, the interaction between microorganisms and the skin/immune system in AD is a very complex network.

### 6.2. The Skin Microbiome in Patients with Seborrheic Dermatitis

**Figure 3:**
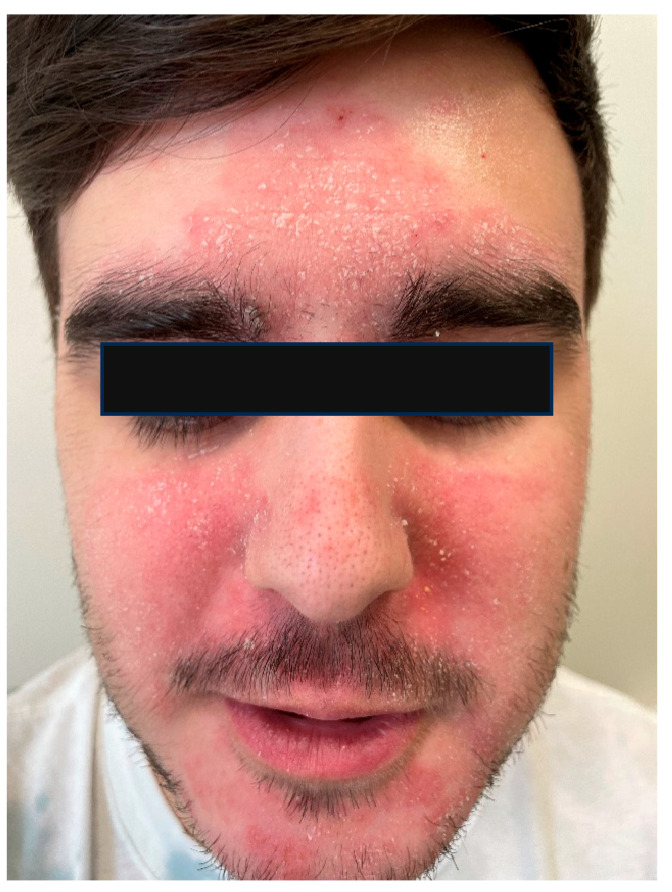
Seborrheic dermatitis—facial lesions.

Seborrheic dermatitis is a chronic inflammatory skin disease, predominantly affecting seborrheic areas, most notably the face (Figure 3). Although the precise pathophysiology remains unclear, current pathogenic theories emphasize the potential role of disrupted interactions between fungal and bacterial communities on the skin surface and their interplay with an impaired skin barrier [44]. Studies of the skin microbiome in patients with seborrheic dermatitis have also shown dysbiosis of the skin affected by the disease compared to healthy areas. As is known, yeasts, especially those of the genus *Malassezia*, play an important role in the etiopathogenesis of seborrheic dermatitis, and their interactions with the skin, bacteria, and the host are important. However, the mechanisms of these interactions are not fully understood.

While the yeast *Malassezia* is considered to be a key factor in disease development, alterations in the relative abundance of bacterial genera such as *Staphylococcus* and *Cutibacterium* compared to *Malassezia*, as well as dysfunction of the skin barrier, may also contribute to the pathogenesis. Some authors propose that intrinsic factors, such as sebum production and the quality of the epidermal barrier, which may be genetically determined, serve as primary triggers for seborrheic dermatitis. These intrinsic factors can be influenced by extrinsic elements, including stress, diet, medications, seasonal changes, and others [71].

Changes in intrinsic factors are thought to create favorable conditions for increased colonization by *Malassezia* yeasts. This, in turn, triggers a Th2 immune response and the secretion of various cytokines (including IL-1α, IL-1β, IL-2, IL-4, IL-6, IL-8, IL-10, IL-12, TNF-α, BDF, IFN-γ, nitric oxide, and histamine), and participates in aryl hydrocarbon receptor-mediated signaling. These processes, together with bacterial dysbiosis, further contribute to skin barrier disruption [72]. Studies investigating the bacterial microbiota of the skin in patients with seborrheic dermatitis have produced inconsistent results. Some authors have reported increased alpha diversity in the skin microbiota of affected patients [73], while others found no significant difference compared to healthy individuals [74]. Nevertheless, most research indicates that the genus *Staphylococcus* is the most prevalent on lesional skin in patients with seborrheic dermatitis [73,74,75,76]. Additionally, there is an increased presence of bacteria from the genera *Streptococcus*, *Acinetobacter*, and *Pseudomonas*, while the abundance of *Cutibacterium* is reduced in comparison to healthy controls [74,75,76,77,78,79]. Thus, in patients with seborrheic dermatitis, a change in the microbiome composition has been confirmed.

### 6.3. The Skin Microbiome in Patients with Acne Vulgaris

Acne vulgaris is a chronic, predominantly facial, inflammatory dermatological condition [25,32,80,81,82]. Concerning the acne pathogenesis, the regulation of various skin cell behaviors by *C. acnes* is an important factor that involves three types of the bacteria, namely I (IA1, IA2, IB, and IC), II, and III. In patients with acne, increased type IA1 abundance and a reduction in type IB and II levels have been found. Pathogenic *C. acnes* target sebocytes, and the highly enriched *C. acnes* in the sebaceous glands/sebocytes stimulate the release of inflammatory factors to promote acne progression, wherein *C. acnes* can induce sebocyte lipogenic activity via the corticotropin-releasing hormone/corticotropin-releasing hormone receiver and the insulin-like growth factor/insulin-like growth factor receiver system [32,80]. The resulting sebum serves as a nutritional substrate for *C. acnes*, maintaining a harmful feedback loop that exacerbates the acne pathology. In keratinocytes, *C. acnes* may directly activate the expression of filaggrin, transglutaminases, and involucrin (through the IGF/IGF-R system), inducing cell proliferation and differentiation. So, *C. acnes* induces the release of inflammatory factors (e.g., human β-defensin 3, IL-1a, IL-8, and matrix metalloproteinases, etc.) by activating receptors (protease-activated receptor-2 (PAR-2) and TLR2/4 in keratinocytes), and also acts through proteases/lipases on keratinocytes to produce hormones and reactive oxygen species (ROS). This process is assisted by the infiltration of inflammatory cells, which exacerbates acne. Also, *C. acnes* specifically triggers dermal preadipocytes to produce cathelicidin through TLR2, which also promotes the acne pathogenesis [80]. Therefore, acne development/progression is associated with sebum degradation, biofilm formation, and inflammation modulation. Bacteria like *C. acnes* break down sebum and release fatty acids, which can be pro-inflammatory and contribute to comedone formation. Also, *C. acnes*, in particular, can form biofilms (bacterial communities encased in a protective matrix) that could contribute to chronic inflammation. Consequent inflammatory responses may further exacerbate skin conditions.

According to research data, an overabundance of *C. acnes* is predominantly linked to acne-prone skin [81,82]. The skin of acne patients mainly contains bacteria of the Firmicutes phylum and the *Staphylococcus* genus, primarily *S. epidermidis* and the Proteobacteria phylum, while the relative abundance of the Actinobacteria phylum is lower than in healthy individuals. According to the relevant research, a loss of bacterial diversity has been observed in acne patients, especially in regard to the diversity of the *C. acnes* phylotype, as well as the dysbiosis of other microbial members of the skin microbiome (which can be impaired/worsened by antibiotic treatment). Otherwise, both healthy individuals and acne patients have similar numbers of *C. acnes* in their follicles, but the skin of affected individuals is overpopulated with specific and particularly virulent strains of *C. acnes*, which can induce a much stronger inflammatory response than the phylotypes associated with healthy skin. In addition, the skin of acne patients has a lower abundance of the genera *Streptococcus*, *Gamella*, *Fusobacterium*, *Granulicatella*, and *Neisseria*, probably due to the relative overgrowth of bacteria of the *Cutibacterium* genus, which limits the growth of other bacteria (which compete for the same ecological space/niche). A positive correlation has also been observed between the prevalence of the genus *Staphylococcus* on the skin and the severity of acne. The most severe stages of the disease were also associated with an increase in the prevalence of bacteria belonging to the genera *Faecalibacterium*, *Klebsiella*, *Odoribacter*, and *Bacteroides*.

There are also other important pathways and factors related to patients with acne. Commensal bacteria, like *S. epidermidis*, mitigate the proliferation of *C. acnes* and moderate the inflammatory cascade [12,80]. Two *S. epidermidis* strains secrete antimicrobial factors (succinic acid, polymorphic toxins), which hamper *C. acnes* growth and suppress keratinocytes-derived inflammatory cytokines, which directly limit acne progression. Thus, *S. epidermidis* also secreted sphingomyelinase, which enhanced keratinocytes’ formation of ceramides and preserved skin homeostasis. Therefore, *S. epidermidis* secretes lipoteichoic acid to induce the upregulation of the microRNA miR-143 and impedes the expression of TLR2 in keratinocytes, effectively inhibiting inflammatory acne progression [30,32,80]. So, a high abundance of *C. acnes* pathogenic strains is crucial for acne development. Pathogenic *C. acnes* strains can grow as biofilms, whose formation is regulated by quorum sensing (QS). Concerning immune cells, *C. acnes* is a potent stimulator of Th17 cells. Its *pathogenic type* IA1 strain drove the differentiation of naive CD4^+^ T cells into Th17 cells (which release IL-17), with the consequent activation of the Th17 immune axis (IL-17 engaged in a feedback loop, compelling CD4^+^ T cells to produce higher levels of IL-17A and IFN-γ, which increased the composite Th17/Th1 immune response). During the Th17 axis activation, mast cells are also activated, resulting in acne initiation. In regard to acne progression, neutrophils predominate and further bolstered Th17 cells (responsible for IL-17 production), leading to encapsulated acne lesions [80].

### 6.4. The Skin Microbiome in Patients with Rosacea

**Figure 4:**
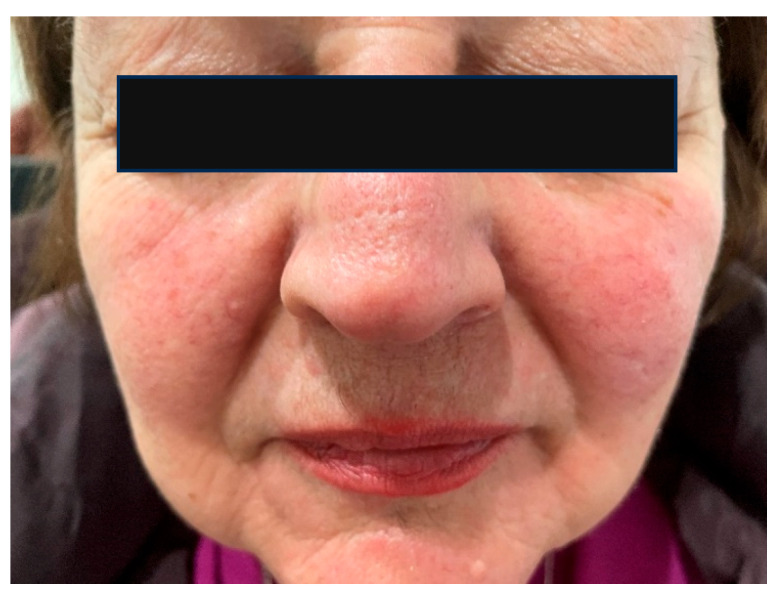
Rosacea—erythematotelangiectatic form.

Rosacea is a chronic inflammatory disorder, characterized by recurrent facial erythema, accompanied by papules, pustules, and telangiectasia, and may also manifest with ocular involvement (Figure 4). The prevalence of rosacea is approximately 5% of the population [83]. Four clinical subtypes are recognized: erythematotelangiectatic rosacea, papulopustular rosacea, phymatous rosacea, and ocular rosacea. Current etiopathogenic theories emphasize the role of the immune system, the generation of reactive oxygen species, abnormal neurovascular signaling, and dysbiosis, all of which promote chronic inflammation and disease progression [84]. In addition to these intrinsic factors, alcohol consumption, spicy foods, and ultraviolet radiation exposure are considered contributory triggers.

Among patients with rosacea, the role of the *Demodex* mite, part of the normal skin microbiome, has been extensively studied regarding its involvement in immune activation and inflammation. *Demodex* infestation is higher in rosacea patients compared to healthy individuals and is considered a potential trigger of the inflammatory process and immune response in these patients [85]. A standardized skin surface biopsy is considered the gold standard technique to evaluate the density of demodex mites. One study reported that the density of *D. folliculorum* on the skin of rosacea patients is up to 5.7 times greater than in healthy controls [85]. This excessive mite burden can lead to follicular and sebaceous gland obstruction, disruption of the skin barrier, tissue damage, and an increase in the expression of TLR2 on keratinocytes and macrophages. Overexpression of TLR2 in rosacea patients may stimulate elevated production of pro-inflammatory cytokines, such as IL-8, IL-1β, and TNF-α, exacerbating the inflammatory response [85,86].

Research on the bacterial microbiota in rosacea, although limited and with inconsistent findings, indicates that *C. acnes* [87,88,89] and *S. epidermidis* [89] are among the most prevalent bacterial species on the skin of affected individuals. Additionally, certain bacteria, such as *Corynebacterium kroppenstedtii,* are found in higher abundance, whereas the genus *Roseomonas* is less represented in rosacea skin [87]. Antibiotic therapy has been shown to reduce rosacea severity and increase the abundance of *Weissella confusa*, a bacterium found in fermented foods and proposed as a probiotic [89]. Thus, in patients with rosacea, according to the relevant research, a higher bacterial diversity of the skin was found compared to healthy individuals, although this difference was not statistically significant. Also, the results of one study found a positive and negative correlation between the severity of rosacea and the abundance of the genera, *Gordonia* and *Geobacillus* (although no statistically significant change in biodiversity was found). In addition, it has been shown that the severity of rosacea increases with age and is associated with a relative decrease in the number of *C. acnes* strains and an increase in the abundance of *Snodgrassella alvi*.

### 6.5. The Skin Microbiome in Patients with Contact Dermatitis

**Figure 5:**
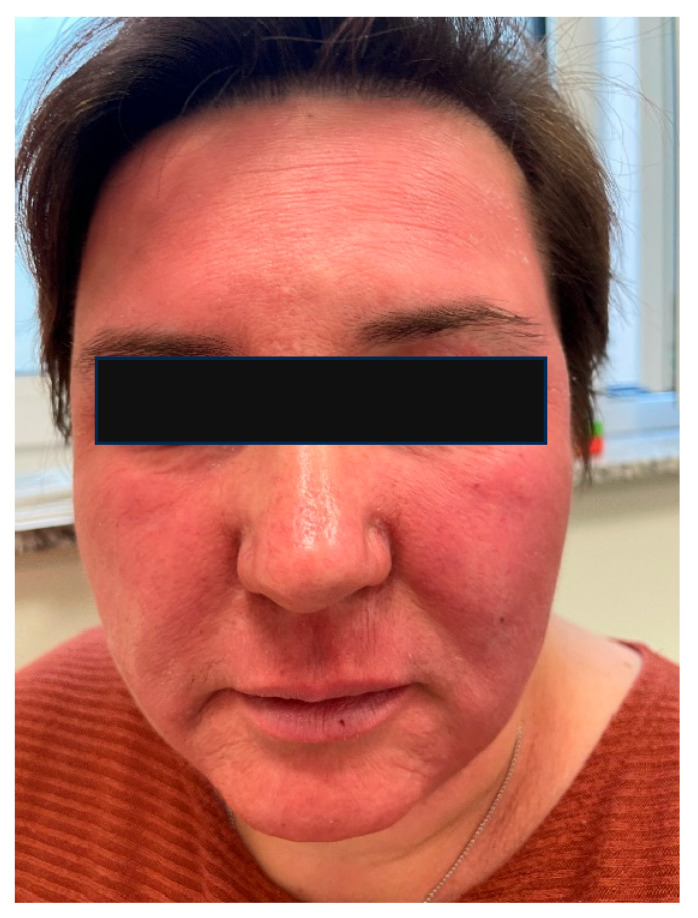
Contact dermatitis—facial lesions.

Contact dermatitis is a common inflammatory skin disease, with a prevalence of up to 15% in the general population, resulting from exposure to contact irritants and allergens (Figure 5). It is the most frequent cause of occupational dermatitis, and the frequent use of cosmetic products represents an additional risk factor for the development of contact dermatitis, especially in women [90,91,92]. Contact dermatitis includes allergic contact dermatitis and irritant contact dermatitis. Allergic contact dermatitis is a type IV delayed hypersensitivity reaction that occurs upon the re-exposure of the skin to an allergen after prior sensitization, whereas irritant contact dermatitis is a nonspecific innate immune response to an irritant without prior sensitization. In regard to the allergic form, during the sensitization phase, small molecules called haptens stimulate epidermal keratinocytes to express adhesion molecules (ICAM-1), pro-inflammatory cytokines (IL-1α, IL-1β, TNF-α, IL-6), and chemokines (IP-10, MCP-1, RANTES, CCL18). This is followed by the processing of neoantigens in Langerhans cells of the epidermis and the migration of these cells to regional lymph nodes, where they present as the antigen to T lymphocytes. Through clonal expansion mediated by cytokines, antigen-specific T lymphocytes are generated, which then travel hematogenously back to the epidermis. Upon re-exposure to the antigen, a faster and more intense inflammatory response occurs [91].

In regard to irritant contact dermatitis, the pathogenesis involves a direct cytotoxic effect through the denaturation of epidermal keratin, the removal of lipids from the skin surface, and damage to cell membranes [90,92]. The acute phase of the disease is characterized by vesicles, macules, and edema, while in the chronic phase, erythema, scaling, and lichenification of the affected skin are observed. Differentiating between the two types of contact dermatitis based on the clinical presentation and history of the patient is often difficult; therefore, patch testing is essential for diagnosing the allergic form [93].

Contact dermatitis can mimic many other diseases and may also occur concurrently with them, which means that taking a thorough medical history of the patient, especially regarding occupational exposure, is crucial for accurate diagnosis. Contact dermatitis is frequently associated with AD. In patients with AD, increased allergen penetration through the skin due to barrier disruption, combined with filaggrin gene mutations, impaired immunoregulatory mechanisms, and the use of topical therapies, may contribute to a higher incidence of contact dermatitis compared to the general population. However, study results on this association are not uniform [94,95,96]. In patients with allergic contact dermatitis of the face and periocular area, the most common contact allergens include metals, cosmetic ingredients (e.g., nail lacquer), preservatives (e.g., benzalkonium chloride), topical antibiotics (e.g., aminoglycosides and bacitracin), fragrances, acrylates, surfactants (e.g., cocamidopropyl betaine), and others [97].

Regarding data on the skin microbiome in patients with contact dermatitis, the data are scarce/rare. Therefore, there are only a few articles that present data on the characteristics of the skin microbiome in patients with contact dermatitis, and the data are mainly related to irritant contact dermatitis (e.g., hand eczema). According to these data, there is an increase in the abundance of *S. aureus* and *Enterococci*, as well as *Erwinia* spp. and *Pseudomonas* spp. However, the data are not consistent, and more research is needed [98,99,100].

### 6.6. The Skin Microbiome in Patients with Periorificial Dermatitis, Including Perioral and Periocular Dermatitis

The skin microbiome in patients with periorificial dermatitis, including perioral/periocular dermatitis, has also been mentioned in the literature, specifically concerning the disruption of the epidermal barrier. Periorificial dermatitis is a relatively common form of chronic inflammatory dermatitis. It predominantly appears around cutaneous orifices, such as the eyes, mouth, nose, and occasionally the genital area [101]. Some authors use the term “periorificial dermatitis” for perioral dermatitis, although the less exact term “periorificial dermatitis” is a more widely used term, which encompasses similar types of lesions at different localizations, like periocular dermatitis and perianal dermatitis (the term “perioral dermatitis” is more precise and suitable for perioral inflammatory lesions). The development of periorificial dermatitis like periocular dermatitis is associated with several factors, including disruption of the skin barrier, activation of the innate immune system, and alterations in the skin microbiome. Increasingly, psychological stress is also recognized as a potential trigger of inflammation, acting either directly through the peripheral nervous system or indirectly via the endocrine and immune systems. The etiopathogenesis of periocular dermatitis, as with other inflammatory skin diseases, involves complex interactions between genetic and environmental factors.

Perioral dermatitis manifests with erythematous lesions (papules, pustules, or vesicles), typically in perioral and perinasal regions, often with concomitant itching or burning sensations [101]. Its pathophysiology is not clearly known, but it is considered a variant of rosacea due to it having a similar histopathology. Perioral dermatitis typically occurs in young adult women, but it can also occur in children. The condition can be triggered by different factors, but occurs mainly due to longer/continuous use of corticosteroids (topical, nasal, and inhaled); the use of cosmetic products; dysfunction of the skin’s epidermal barrier (such as a contact reaction); the potential influence of allergens/irritants; the presence of microorganisms (e.g., *Demodex* spp., *Candida albicans*, bacteria from the genus *Fusobacterium*); hormonal changes (due to oral contraceptive use, pregnancy, and premenstrual flares); and an atopic predisposition [102,103]. Perioral dermatitis lesions may worsen or be related to mineral or vitamin deficiencies (e.g., a zinc deficiency), occlusive emollient use (overhydration); the use of sunscreen; or prolonged exposure to ultraviolet radiation, or other environmental factors like heat or wind [101,104].

According to the data on the skin microbiome in perioral and periocular regions, bacteria of the genera *Streptococcus* and *Rothia* predominate on the skin of the perioral area in healthy infants. Research on the bacterial microbiome of healthy periocular skin is limited, but has shown that the dominant bacterial phyla in the periocular region are Actinobacteria, followed by Firmicutes, Proteobacteria, and Bacteroidota, which corresponds with other seborrheic areas of the skin [8,29]. Another common finding in regard to the skin of the periocular region is the *Demodex* mite, which is sometimes observed in healthy individuals, but is even more common in patients with blepharitis, wherein its role is still insufficiently understood.

In addition, demodicosis, which involves a variety of dermatoses caused by *Demodex* mites, is also possible. These mites are considered commensal organisms of the pilosebaceous units of seborrheic localizations like the face, scalp, and chest. However, *D. folliculorum* and *D. brevis* have been implicated in papulopustular rosacea, perioral dermatitis, and other papulopustular eruptions. However, they are still often overlooked during the differential diagnosis of chronic, pruritic eruptions of sebaceous skin, but the increasing use of dermatoscopy in clinical practice/examinations offers a potential new option for the rapid diagnosis of this infestation [36].

## 7. A Review of the Studies on the Role of the Skin Microbiome in Facial Dermatoses

The development of molecular methods for studying microorganisms that inhabit the human body and skin has brought the skin microbiome into the spotlight as a potential factor in disease development, a diagnostic marker, and a target for new therapeutic approaches. Therefore, recent research on the pathogenesis of skin diseases emphasizes the need to understand the complex interactions between skin components and the microbiome, which includes bacteria, fungi, viruses, and archaea residing on the skin [11]. However, studying the skin microbiome is particularly challenging because the amount of DNA collected from the skin surface is extremely low compared to, for example, the quantity of DNA obtained from stool samples [18]. According to the relevant research, the results from microbiome analysis using skin swab sampling and stripping methods provide useful data, while the results obtained using biopsy techniques offer more detailed information about the microbiome composition, especially studies of dermal diseases, such as psoriasis [105,106,107].

Research comparing the microbiome of different facial areas of healthy skin has revealed that the lips and eyelids, i.e., periorificial regions, are characterized by a distinct microbiome composition and greater biodiversity compared to other facial areas [108]. On the eyelid skin, a higher abundance of bacteria belonging to the genus *Staphylococcus* was found compared to other sites, such as the forehead, cheeks, and chin, along with differences in alpha and beta diversity. These differences in the microbiome composition of periorificial areas arise from specific topographical relationships, due to the transition from skin to conjunctiva or oral mucosa, and from the use of cosmetic products that may influence microbiome changes. According to recent findings by Ferček et al. (partially consistent with earlier studies), the microbiome of the eyelid skin is specific and differs in composition from other seborrheic skin areas [109]. Robert et al. investigated differences in the bacterial microbiome according to sex by analyzing cheek samples from adult subjects [110]. Based on alpha diversity analysis, they found lower biodiversity in male subjects and observed a separation between male and female subjects based on beta diversity. The higher biodiversity in women is explained by their thinner skin, more acidic skin pH, reduced sebum and sweat production, and more frequent use of cosmetic products [108].

The role of the bacterial species *S. aureus* and *S. epidermidis* in the pathogenesis of AD has been extensively studied [59,67,111]. Skin dysbiosis in patients with AD is characterized by an increase in the abundance of *Staphylococcus* species and a simultaneous decrease in other potentially beneficial microbial species. It is known that *Staphylococcus* bacteria secrete AMPs that can reduce the presence of other bacterial genera, such as *Corynebacterium*, *Streptococcus*, *Lactobacillus*, and *Finegoldia* [59,112]. The reasons why the skin of AD patients is more conducive to colonization by *S. aureus* are not yet fully understood. These bacteria initially colonize the stratum corneum and secrete toxins and proteases that can damage it, thereby impairing the effectiveness of the epidermal barrier, a process further exacerbated by a diminished innate immune response. *S. aureus* expresses superantigens that activate polyclonal T lymphocytes and trigger inflammation. They also act as allergens, inducing IgE-mediated responses following histamine release from mast cells and basophils, and can promote inflammation by releasing phenol-soluble modulins that stimulate keratinocytes to release pro-inflammatory cytokines, such as IL-8 and IL-1β, as well as inducing mast cell degranulation [113].

In patients with rosacea, a decrease in the abundance of the genus *Cutibacterium* has been observed compared to healthy controls, specifically involving the species *C. acnes* and *Cutibacterium avidum* [109,114,115]. The role of *Cutibacterium*, especially *C. acnes*, in the pathogenesis of inflammatory dermatoses has been most extensively studied in patients with acne [116,117]. Despite occasional similarities in the clinical features between rosacea and acne, their clinical courses and characteristics differ; however, *C. acnes* has emerged as a potential causative factor in the development of inflammation in both diseases [116]. Recent studies have identified different phylotypes of *C. acnes*, including IA, IB, II, and III, with a further subdivision of phylotype I into IA1, IA2, IB, and IC. Phylotype IA1 is predominantly found in patients with acne, whereas phylotypes IA2, IB, II, and III are mostly present in healthy individuals, where they contribute to maintaining healthy skin homeostasis and play a protective role against inflammatory disease development [116]. A higher prevalence of certain phylotypes in acne patients may lead to disease development, while in rosacea patients, the absence of protective defense mechanisms is likely important in the disease pathogenesis. *C. acnes* not only participate in lipid metabolism, but also secrete lipases, metalloproteases, and porphyrins, which, in the presence of oxygen, can generate oxidative stress that damages keratinocytes. Additionally, *C. acnes* can activate the innate immune system via the NLRP3 inflammasome, promoting inflammation through the activation of caspase-1 and the secretion of IL-1β and IL-18, cytokines responsible, for example, for the development of papules in acne patients [117].

The limited studies describing the skin microbiota of the eyelids have not shown significant differences in composition compared to other seborrheic skin areas [8,29]. According to one study, the most abundant phylum on the eyelid skin is Actinobacteria, followed by Firmicutes, Proteobacteria, and Bacteroidota. Another study reported differences in skin microbiome composition between younger and older individuals: in people aged ≤35 years, the genera *Cutibacterium* and *Staphylococcus* are most prevalent, whereas in those over 65 years, *Corynebacterium* and members of the Neisseriaceae family predominate [35]. Changes in the bacterial skin microbiome according to age and sex have also been investigated. Recent research identified both positive and negative trends in terms of biodiversity, which was slightly higher in patients with periocular dermatitis who were over 50 years old compared to those under 50, based on the alpha diversity indices [109]. In healthy individuals, a decline in biodiversity was observed in those over 50 years of age. The data on sex-related differences in regard to biodiversity are inconsistent, with some studies reporting higher biodiversity in males and others in females, while recent studies have demonstrated significantly greater alpha diversity in older individuals [109,118].

It is important to mention the conflicting research findings. While some research results show a clear link between specific microbiome alterations and various dermatoses, other studies highlight the complexity and variability of the obtained findings, with influences identified from some related factors, like host genetics, environmental influences, and treatment interventions. Also, there is variable disease presentation, as dermatoses may manifest differently in individuals, leading to variations in the affected skin areas and the extent of the impaired skin barrier. In addition, there are some confounding factors like genetics, age, immune status, and lifestyle features (e.g., smoking, diet), which may also influence the skin microbiome composition and contribute to conflicting findings. In addition, there are methodological differences among the studies, which use different techniques for microbiome analysis (culture-based methods or molecular-based methods), which may lead to discrepancies in the results. Also, there is an influence from any previous treatments participants may have undergone, like antibiotics, phototherapy, and even topical emollients, which may change the skin microbiome. In addition, skin dysbiosis may be both a consequence, as well as a cause, of disease. Thus, it may be pertinent to mention the complexity of the skin–gut axis and the association between the skin microbiome and the gut microbiome, according to which changes in one may impact the other, further complicating the study results. Finally, the microbiome has a dynamic nature, it changes over time and is influenced by many factors (e.g., treatment interventions, disease progression, and environmental conditions, etc.).

## 8. New Treatment Options Based on the Skin Microbiome and Perspectives in This Field

Gathering knowledge about the skin microbiome, as well as the gut microbiome, is important for developing new treatment modalities for inflammatory skin diseases that have traditionally been managed using antibiotics, corticosteroids, laser therapy, and other conventional treatments. These new approaches include modulation of the gut and skin microbiomes through the systemic and topical use of prebiotics and probiotics [119,120,121,122]. Probiotics are live microorganisms that confer health benefits to the host, while prebiotics are substrates (e.g., insoluble fibers, inulin, fructooligosaccharides) that promote the survival and growth of probiotics. Probiotics (found in foods like yogurt, kefir, sauerkraut, and kimchi) introduce beneficial bacteria to the gut/skin, promoting a balanced microbiome. Prebiotics, as non-digestible fibers, which act as food for beneficial bacteria, may also be useful. The combination of probiotics and prebiotics is termed synbiotics.

Dietary choices can also be considered as a means of microbiome modulation. Historical changes to dietary habits, particularly the so-called “Western diet”, characterized by a high intake of processed foods, saturated fats, and refined sugars, have contributed to an increase in the incidence of diseases, such as irritable bowel syndrome, cardiovascular diseases, metabolic disorders, obesity, and type 2 diabetes [119]. Conversely, diets rich in highly digestible foods (which reduce indigestible substances in the gut lumen and the overgrowth of certain bacterial species) and diets high in hydrolyzed proteins have been shown to reduce inflammatory responses [120,121,122]. Therefore, such diets are recommended for the prevention and treatment of these diseases, as well as inflammatory skin conditions like acne.

All of the previously mentioned data on the skin microbiome may be related to its therapeutic potential for patients, so understanding the interplay between hormones and the microbiome is crucial for developing targeted therapies for specific dermatoses (Table 1). For example, probiotic supplementation (with specific bacterial strains) may potentially help restore balance to the skin microbiome and address certain skin conditions. Also, a balanced diet can significantly impact the skin microbiome. Incorporating foods rich in probiotics, prebiotics, and antioxidants can promote a healthy gut/skin microbiome and potentially act on inflammation, improving specific dermatoses. On the other hand, diets high in processed foods, sugar, and unhealthy fats may negatively impact the skin microbiome, potentially worsening skin conditions.

The effects of oral probiotics have been investigated in the treatment of skin diseases, diabetes, obesity, autism, and depression, whereas their topical application has been studied in patients with AD, acne, and psoriasis [120]. According to conducted studies, the topical application of solutions containing gram-negative bacteria, such as *Roseomonas mucosa* or *Lactobacillus johnsonii,* can alter the microbiome composition in patients with AD, contribute to clinical improvements, reduce disease severity, and decrease the need for corticosteroid use [123,124]. However, most of the topical probiotic preparations currently used are personal care products, such as creams and serums, for skin rejuvenation. An example is a probiotic cream containing *Nitrosomonas eutropha*, which, according to research by Notay et al., effectively reduces wrinkles and hyperpigmentation [125]. Thus, topical therapies that interact with skin microbiomes, such as those containing prebiotics, probiotics, or postbiotics, are increasingly recognized for their potential in managing various dermatoses. These therapy options aim to modulate the skin’s microbial community, influencing skin health and potentially alleviating the symptoms of conditions like acne, AD, and psoriasis. Also important are antioxidants, as they protect the skin from free radical-induced skin damage, potentially improving skin health. Also, staying adequately hydrated is important for a healthy skin microbiome.

Finally, in this field, fecal microbiome transplantation (FMT) is mentioned as a therapeutic option for treating recurrent post-antimicrobial diarrhea caused by *Clostridium difficile* that is unresponsive to antibiotic therapy, aiming to restore a healthy gut bacterial microbiome and have a positive impact on various conditions, including dermatoses. Transplantation is performed via endoscopy, enema, or oral capsule ingestion. Limitations of this therapy include a lack of quality control and insufficient understanding of microorganism–host interactions to ensure safe applications [126]. Although these therapeutic options are not yet widely used, the initial research results are encouraging regarding their efficacy.

In conclusion, the skin microbiome plays a complex role in various dermatoses, including facial dermatoses, wherein microbiome features and changes are associated with many dermatoses. Establishing definitive causal relationships requires further investigations, taking into account the many related and confounding factors and the dynamic nature of the microbiome. It should be emphasized that the skin microbiome influences skin health and immune system regulation; it has a key role in regulating the immune system, supporting the maintenance of skin health, and the prevention of infections. It is important to remember that microbes may impact the skin’s barrier function, including its ability to protect the body against external factors and retain moisture. Dysbiosis can lead to inflammation and dermatoses, so it is useful to be able to predict skin conditions, whereby microbiome profiling plays an important role by analyzing the types and amounts of skin microbes. In other words, early detection and intervention can potentially prevent or mitigate the severity of dermatoses. All this is important for the development of personalized treatments, as well as the targeting of specific imbalances.

## 9. Conclusions

Based on the abundant evidence of skin dysbiosis and specific deviations in the composition of the skin microbiome in patients with certain facial dermatoses, future research on the skin microbiome in patients with certain dermatoses compared to healthy individuals could play an important role in gaining additional knowledge about the healthy and altered skin microbiome, as well as in determining the presence of dysbiosis in individual patients. Emerging evidence indicates that the skin microbiome contributes to maintaining skin homeostasis and may influence inflammatory skin disorders. Therefore, more research and knowledge about the specificities of the microbiome in patients with certain diseases and the application of this knowledge in regard to the treatment of such patients are expected to be generated, primarily involving the design of new therapeutic options.

## Figures and Tables

**Table 1:** A comparison of microbiome alterations across different dermatoses.

	Atopic Dermatitis	Seborrheic Dermatitis	Acne Vulgaris	Rosacea	Contact Dermatitis
**↑** **Increased**	*Staphylococcus* spp. *Staphylococcus aureus* *Staphylococcus epidermidis*	*Malassezia spp.* *Staphylococcus* spp. *Staphylococcus epidermidis* *Streptococcus* spp. *Pseudomonas* spp. *Acinetobacter*	*Cutibacterium acnes (IA1)* *Cutibacterium granulosum* *Staphylococcus epidermidis* *Proteobacteria* *Firmicutes* *Streptococcus spp.* *Malassezia spp.*	*Demodex folliculorum* *Staphylococcus epidermidis* *Corynebacterium kroppenstedtii*	*Staphylococcus aureus* *Enterococcus* spp. *Erwinia* spp. *Pseudomonas* spp.
**Decreased** **↓**	*Streptococcus* spp. *Cutibacterium* spp. *Corynebacterium* spp.	*Cutibacterium* spp.	*Actinobacteria*	*Roseomonas* spp. *Cutibacterium acnes* *Cutibacterium avidum*	*Clostridium* spp. *Actinomyces* spp. *Staphylococcus epidermidis* *Bifidobacterium* spp. *Anaerococcus* spp. *Staphylococcus haemolyticus*
